# Analysis of Social and Genetic Factors Influencing Heterosexual Transmission of HIV within Serodiscordant Couples in the Henan Cohort

**DOI:** 10.1371/journal.pone.0129979

**Published:** 2015-06-11

**Authors:** Qian Zhu, Peng Zhu, Yilei Zhang, Jie Li, Xuejun Ma, Ning Li, Qi Wang, Xiujuan Xue, Le Luo, Zizhao Li, Huijun Z. Ring, Brian Z. Ring, Li Su

**Affiliations:** 1 Institute for AIDS/STD Prevention and Control, Henan Center for Disease Prevention and Control, Zhengzhou, China; 2 Key Laboratory of Molecular Biophysics of Ministry of Education, School of Life Science and Technology, Huazhong University of Science and Technology, Wuhan, China; 3 State Key Laboratory for Molecular Virology and Genetic Engineering, National Institute for Viral Disease Control and Prevention, Chinese Center for Disease Control and Prevention, Beijing, China; 4 Health Department of Henan Province, Medical Science and Education Building, Zhengzhou, China; 5 Institute for Genomic and Personalized Medicine, School of Life Science and Technology, Huazhong University of Science and Technology, Wuhan, China; University of Texas Health Science Center San Antonio Texas, UNITED STATES

## Abstract

There is considerable variability between individuals in susceptibility to infection by human immunodeficiency virus (HIV). Many social, clinical and genetic factors are known to contribute to the likelihood of HIV transmission, but there is little consensus on the relative importance and potential interaction of these factors. Additionally, recent studies of several variants in chemokine receptors have identified alleles that may be predictive of HIV transmission and disease progression; however the strengths and directions of the associations of these genetic markers with HIV transmission have markedly varied between studies. To better identify factors that predict HIV transmission in a Chinese population, 180 cohabiting serodiscordant couples were enrolled for study by the Henan Center for Disease Prevention and Control, and transmission and progression of HIV infection were regularly measured. We found that anti-retroviral therapy, education level, and condom use were the most significant factors in determining likelihood of HIV transmission in this study. We also assessed ten variants in three genes (*CXCL12*, *CCR2*, and *CCR5*) that have been shown to influence HIV transmission. We found two tightly linked variants in *CCR2* and *CCR5*, rs1799864 and rs1800024, have a significant positive association with transmission as recessive models (OR>10, *P* value=0.011). Mixed effects models showed that these genetic variants both retained significance when assessed with either treatment or condom use. These markers of transmission susceptibility may therefore serve to help stratify individuals by risk for HIV transmission.

## Introduction

Human immunodeficiency virus infection/ acquired immunodeficiency syndrome (HIV/AIDS) has the highest mortality among infectious diseases in China and has become a public health crisis. In response to this health challenge, since 2003 the national government has offered a free antiretroviral treatment and prevention program. This effort was piloted in 2002 on a large Henan cohort and has led to a significant decrease in AIDS related mortality in the region. However, a 2011 joint assessment by the World Health Organization, UNAIDS, and the Chinese Ministry of Health found there remained about 780 000 people infected with HIV in China, with approximately 154,000 cases of AIDS. Within this cohort, more than 60% of transmissions occurred heterosexually, the remaining transmissions were due to intravenous drug use, blood transfusions, mother to child transmission, and male-to-male sexual contact [1]. Therefore cohabiting serodiscordant couples (HIV infected individuals with uninfected partners of opposite sex) are at an especial high risk of transmission.

Genetic variations have been shown to be associated with HIV susceptibility and AIDS progression [2,3]. In particular, chemokine (C-C motif) receptor 5 (CCR5), a coreceptor HIV-1 uses in entering its target cells, plays an important role in HIV infection [4]. Homozygosity for the Δ32 (rs333) allele of *CCR5* was found to provide protection against infection with CCR5-tropic HIV clades [5,6], and homozygous “A” alleles in the 3’ untranslated region of *CXCL12* (C-X-C motif chemokine 12, also known as stromal cell-derived factor 1, *SDF-1*) was discovered to delay AIDS progression [7]. Interestingly, a study on one patient showed that HIV-1 infected patient treated with *CCR5* Δ32/Δ32 stem cell transplantation reconstituted CD4^+^ T cell and remained without viral rebound after treatment [8,9]. However, a survey in a Chinese population found no homozygous *CCR5* Δ32 individuals, and only about 0.36% (5 of 1406) were carriers of the *CCR5* Δ32 allele [10,11]. Other *CCR5* alleles and one *CCR2* variant have shown varying levels of association with risk of HIV transmission and disease progression [12]. Further investigations within ethnically or genetically defined populations are needed to better identify clinically relevant markers.

Because of the high HIV infection level, there is a national initiative in China to study how social, clinical and phamacogenetic markers can be used in predicting infection risk and treatment response. Four counties in Henan province (Shenqiu, Shangcai, Weishi and Queshan counties) with high prevalence of HIV infection caused by HIV infected former plasma donation in the 1990s [13], have been the focus of national efforts to control HIV transmission. Cohabiting serodiscordant couples were enrolled a follow-up investigation by Henan CDC from 2005 to the present day in a cohort study. The objectives of this study were to determine which factors affect HIV transmission from seropositive individuals (index partners) to uninfected partners (exposed partners), and to study any interactions between genetic and social factors predictive of transmission. Transmission and progression of HIV infection were regularly measured, and potentially relevant sociological factors were queried via surveys. Additionally, several genetic markers were assessed, and the associations of these factors with transmission were determined.

## Materials and Methods

### Study Subjects

All cohabiting couples of discordant HIV serostatus living in four areas (Shenqiu, Shangcai, Weishi and Queshan counties) with high prevalence of HIV infection in Henan province were enrolled in an investigation by the Henan CDC from 2005 to 2010. HIV serostatus of each couple was measured twice every year by local CDC and data were collected and inputted to database. Selection criteria of participants were: 1) subjects were permanent residents or living in the four areas for more than six months; 2) the subjects were married to each other at the time of infection and had remained married until surveyed; 3) relevant data was available from the Henan CDC; 4) exposed partner was seronegative at initiation of investigation; 5) exposed partner didn’t have any other known behavior that could increase risk of infection (such as intravenous drug use, plasma donation, having multiple sex partners, etc). Seroconverted exposed partners meeting these criteria were defined as transmission couples, and compliant non-transmission couples were selected randomly from the seronegative exposed partners. The final study consisted of 87 transmission and 93 non-transmission couples. All the participants provided written informed consent and this study was approved by the Henan Province Health Department and the Henan CDC ethics committee (reference number HNCDC-2010-15). A questionnaire was used to collect information on participants’ personal characteristics, behavior and medical history. Condom use was queried and as coded as 1 = every sexual encounter, 2 = frequent use (the majority of sexual encounters), 3 = infrequent use (minority of sexual encounters), and 4 = never used.

### Genomic DNA Purification and Genotyping

Genomic DNA was purified from the exposed partner’s whole blood samples obtained from Henan CDC, then quantified and diluted as described previously [14]. Based on a literature review, ten SNPs: *CXCL12* c.*519G>A (rs1801157), *CCR2-CCR5* haplotype (rs1799864, rs2856758, rs2734648, rs1799987, rs1799988, rs41469351, rs1800023, rs1800024 and rs333) were selected to genotype. Briefly, genomic DNA samples were amplified for each SNP in 96-well PCR plates on PCR thermal cycler (Bioer, Hangzhou, China). Then PCR products were purified with magnetic beads (GenMag Biotechnology Co., Ltd., Beijing, China) using Biomek FX^P^ laboratory automation workstation (Beckman coulter). Purified PCR products were sequenced by ABI 3730xl. Primers for PCR and sequencing are listed in S1 Table.

### Statistical Analysis

Strength of association, *P*-values, odds ratios (ORs) and 95% confidence intervals (95% CIs) for association between these genotypes and transmission status were estimated using the Fisher exact test for gender, Student’s *t*-test for age, viral load and frequency of sexual intercourse, and Cochran Armitage tests for ordered categorical data, such as education level and frequency of condom use. Logistic regression and generalized linear mixed models were used when assessing independence of multiple covariates. For the genetic analyses the common allele was used as the reference category. Statistical analysis was performed with R [15] and the Cochran Armitage tests with the coin package [16]. For haplotype analysis, maximum likelihood estimates of trait associations with SNP haplotypes were measured using the method of Burkett *et al*. with the hapassoc package [17]. Linkage analysis of the CCR2-CCR5 alleles was performed with Haploview [18].

## Results

### Participants’ Demographic Characteristics

**Fig 1:**
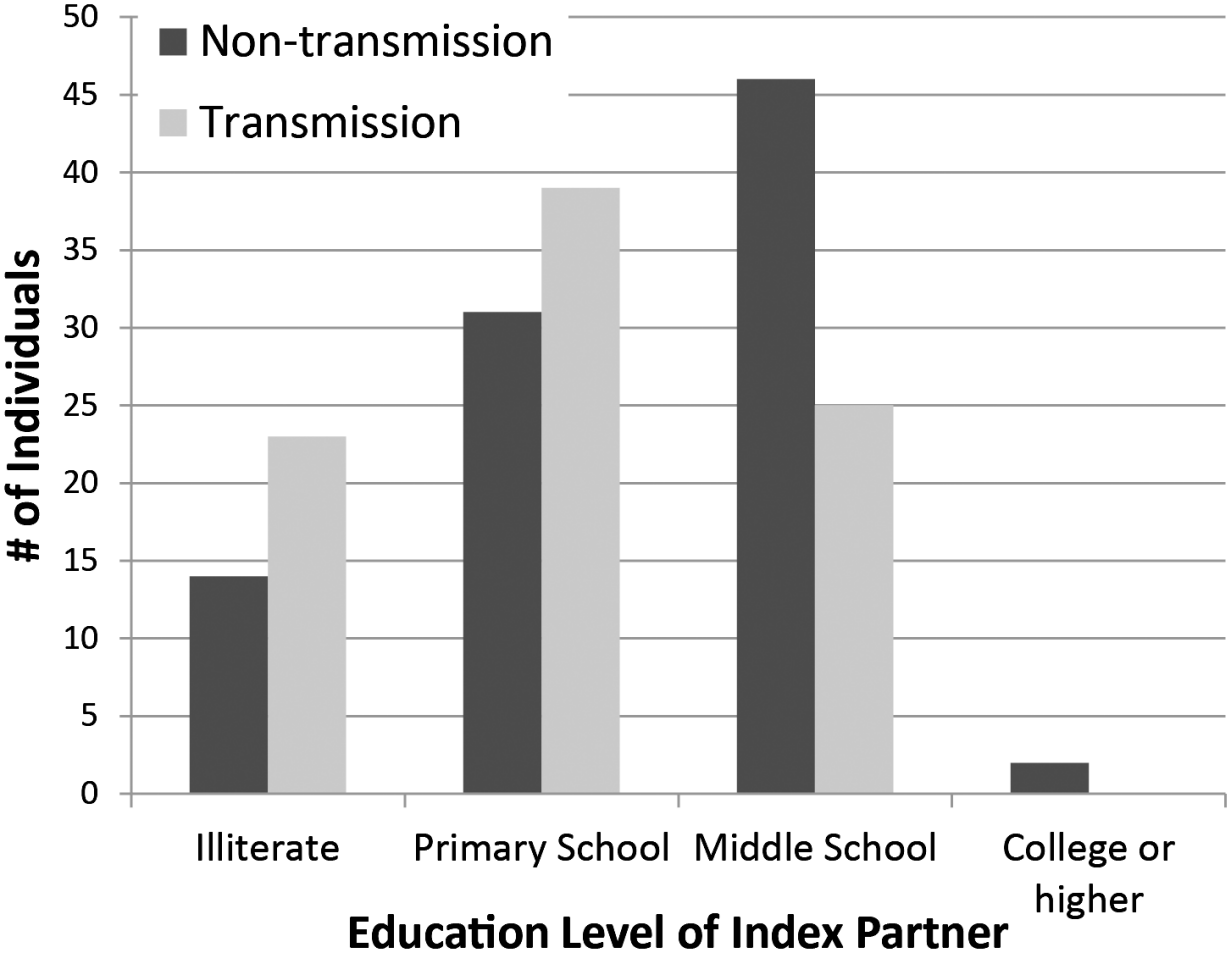
Education level and numbers of HIV transmission cases.

For these analyses, 87 transmissions and 93 non-transmissions couples met the participation criteria. Cohort characteristics are shown in Table 1. The male/female ratios of index partners are 42/45 and 53/40 for the transmission group and non-transmission group, respectively. 79 of 87 and 72 of 93 index partners received anti-retroviral therapy (ART) in the transmission and non-transmission groups, Fisher exact test showed that reception of ART positively associated with risk of HIV transmission within couples (*P* = 0.025). Viral load did not show a significant association with transmission (*P* = 0.496). In the transmission couples, 23 and 13 of index/exposed partners were illiterate, 39 and 38 of index/exposed partners received only primary education, 25 and 36 of index/exposed partners received secondary or higher education. However in the non-transmission group, 14 and 14 of index/exposed partners were illiterate, 31 and 29 index/exposed partners received only a primary education, and 48 and 50 index/exposed partners received secondary or higher education (Fig 1). Education level of index partners was found to be significantly (*P* = 0.002) negatively associated with transmission status using the Cochran-Armitage test, while no significance (*P* = 0.255) was found between education level of exposed partners and transmission status. Age distribution of exposed partners was not found to be significantly associated with transmission status using a Fisher exact test. The most significant factor associated with transmission is condom use, with both the frequency of usage (as related by either partner) before and after the diagnosis of the index partner for HIV infection having a significant negative association with transmission. Change in condom use (measured as ‘frequency of use after diagnosis’–‘frequency of use before diagnosis’) was of even greater significance, with increased use of condoms after diagnosis of the index partner with HIV infection being strongly associated with decreased occurrence of transmission (Table 2).

**Table 1:** Cohort characteristics.

	Transmission	Nontransmission	*P*
**Number of couples**	87	93	
**Gender of index partners**			0.307^a^
Male	42 (48.28%)	53 (56.99%)	
Female	45 (51.72%)	40 (43.01%)	
**Antiretroviral therapy**			0.025^a^
Did not receive	8 (9.20%)	21 (22.58%)	
Received	79 (90.80%)	72 (77.42%)	
**Education level of index partners**			0.002^b^
Illiteracy	23 (26.44%)	14 (15.05%)	
Primary education	39 (44.83%)	31 (33.33%)	
Secondary education (or higher)	25 (28.74%)	48 (51.61%)	
**Education level of exposed partners**			0.255^b^
Illiteracy	13 (14.94%)	14 (15.05%)	
Primary education	38 (43.68%)	29 (31.18%)	
Secondary education (or higher)	36 (41.38%)	50 (53.76%)	
**Age of index partners (years, at diagnosis)**			0.473^c^
20–29	15 (17.24%)	12 (12.90%)	
30–39	32 (36.78%)	53 (56.99%)	
40–49	29 (33.33%)	20 (21.51%)	
>50	11 (12.64%)	8 (8.60%)	
**Age of exposed partners (years, at diagnosis)**			0.249^c^
20–29	13 (14.94%)	14 (15.05%)	
30–39	34 (39.08%)	51 (54.84%)	
40–49	32 (36.78%)	22 (23.66%)	
>50	8 (9.20%)	6 (6.45%)	
**Viral load of Index partners (RNA copies/mL)**	11063	17956	0.496^c^

^a^ Fisher exact test.

^b^ Cochran-Armitage test.

^c^ Student’s *t-*test.

**Table 2:** Cohort behavior characteristics.

	Transmission	Nontransmission	*P*
**Condom use before Index diagnosed** ^**i**^			<0.001^b^
Every time	14 (16.09%)	4 (4.30%)	
Usually (>1/2)	5 (5.75%)	5 (5.38%)	
Infrequent (<1/2)	2 (2.30%)	15 (16.13%)	
Never	37 (42.53%)	65 (69.89%)	
No answer	29 (33.33%)	4 (4.30%)	
**Condom use after Index diagnosed** ^**i**^			0.012^b^
Every time	46 (52.87%)	71 (76.34%)	
Usually (>1/2)	16 (18.39%)	3 (3.23%)	
Infrequent (<1/2)	7 (8.05%)	6 (6.45%)	
Never	9 (10.34%)	5 (5.38%)	
No answer	9 (10.34%)	8 (8.60%)	
**Condom use before Index diagnosed** ^**e**^			0.003^b^
Every time	6 (6.9%)	4 (4.3%)	
Usually (>1/2)	2 (2.3%)	4 (4.3%)	
Infrequent (<1/2)	0 (0%)	15 (16.13%)	
Never	37 (42.53%)	61 (65.59%)	
No answer	42 (48.28%)	9 (9.68%)	
**Condom use after Index diagnosed** ^**e**^			<0.001^b^
Every time	42 (48.28%)	72 (77.42%)	
Usually (>1/2)	17 (19.54%)	3 (3.23%)	
Infrequent (<1/2)	10 (11.49%)	4 (4.3%)	
Never	7 (8.05%)	8 (8.6%)	
No answer	11 (12.64%)	6 (6.45%)	
**Change in condom use (average)**	-1.2	-2.2	<0.001^b^
**Frequency of sexual intercourse before diagnosis (per month)** ^**i**^	4.51	5.06	0.085^c^
**Frequency of sexual intercourse after diagnosis (per month)** ^**i**^	2.93	2.97	0.889^c^
**Frequency of sexual intercourse before diagnosis (per month)** ^**e**^	4.34	5.03	0.035^c^
**Frequency of sexual intercourse after diagnosis (per month)** ^**e**^	2.91	3.05	0.598^c^

^b^ Cochran-Armitage test.

^c^ Student’s *t-*test.

^d^ Change in condom use was measured as ‘frequency of use after diagnosis—‘frequency of use before diagnosis, with use coded as: 1 = every sexual encounter, 2 = frequent use (the majority of sexual encounters), 3 = infrequent use (minority of sexual encounters), and 4 = never used.

^e^ Data according to surveys of exposed partners.

^i^ Data according to surveys of index partners.

### Genetic Variants Associated with HIV Seroconversion

**Fig 2:**
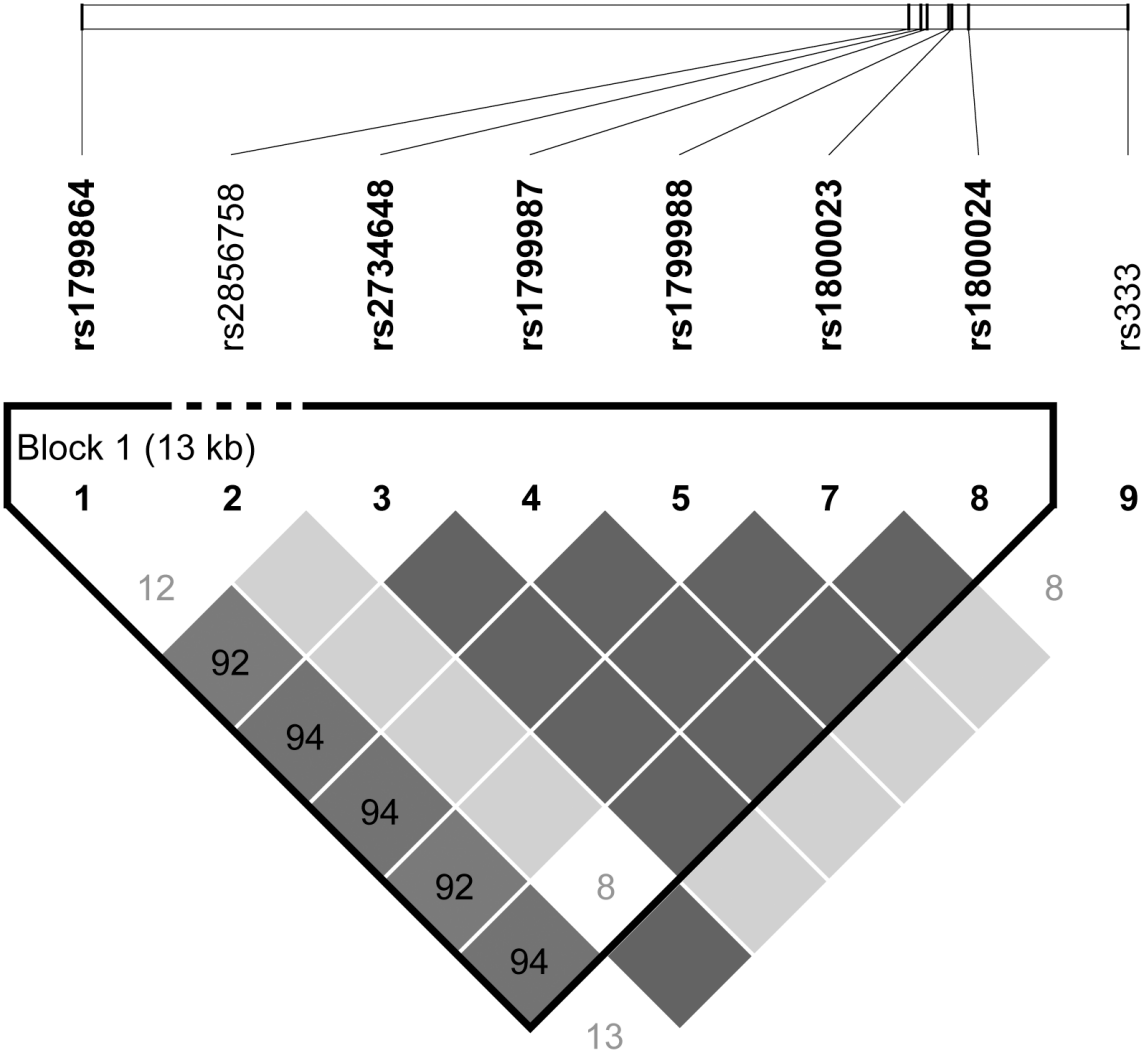
Linkage disequilibrium analysis of the *CCR2* and *CCR*5 alleles included in this study.

All genotypes were assessed as recessive, dominant, and additive models. S1 Fig shows the variants studied and their relative positions in the genes *CCR2*, *CCR5* and *CXCL12*. The *CCR5* variants rs2856758 and rs333 did not occur as homozygous mutant in any individual in this study, and *CCR5* rs41469351 was only present as homozygous wild type (Table 3). LD analysis shows that several of the *CCR2-CCR5* alleles are tightly linked (Fig 2). The *CCR2* variant rs1799864 and *CCR5* rs1800024 showed a significant positive association (OR>10, *P* = 0.011) with transmission as recessive models (Table 4). No other model for other variants showed significance with transmission. Analysis was performed separately in the treated and untreated patients, however no untreated individuals who were homozygous mutant at rs1799864 and rs1800024 were found in this study. This subset analysis did not identify any additional alleles with significant association with transmission. Ten *CCR2-CCR5* haplotypes were identified in this population, all haplotypes were assessed for association with other clinical variables when their frequency in the cohort was greater than 5%, and none showed a significant association with transmission (Table 5). Haplotypes are presented as alleles in the order: rs1799864, rs2856758, rs2734648, rs1799987, rs1799988, rs41469351, rs1800023, rs1800024, rs333 (the rs333 *CCR5* Δ32 genotype was coded as I (insertion)/D (deletion)), and by a previously proposed phylogenetic-based nomenclature [19].

**Table 3:** Distribution of selected SNPs.

Gene	SNP	Allele	Frequency	Genotype	Subjects (%)	*P*(HWE)
*CXCL12*	rs1801157	G	0.825	GG	122 (67.78%)	0.8017
		A	0.175	GA	52 (28.89%)	
				AA	6 (3.33%)	
*CCR2*	rs1799864	G	0.7861	GG	109 (60.56%)	0.3832
		A	0.2139	GA	65 (36.11%)	
				AA	6 (3.33%)	
*CCR5*	rs2856758	A	0.9944	AA	178 (98.89%)	1.0000
		G	0.0056	AG	2 (1.11%)	
				GG	0	
	rs2734648	G	0.4861	GG	37 (20.56%)	0.1351
		T	0.5139	GT	101 (56.11%)	
				TT	42 (23.33%)	
	rs1799987^a^	G	0.6006	GG	58 (32.40%)	0.0608
		A	0.3994	GA	99 (55.31%)	
				AA	22 (12.29%)	
	rs1799988	T	0.6	TT	59 (32.78%)	0.0883
		C	0.4	TC	98 (54.44%)	
				CC	23 (12.78%)	
	rs41469351	C	1	CC	180 (100.00%)	NA
		T	0	CT	0	
				TT	0	
	rs1800023^a^	A	0.4832	AA	36 (20.11%)	0.1006
		G	0.5168	AG	101 (56.42%)	
				GG	42 (23.46%)	
	rs1800024^b^	C	0.7725	CC	103 (57.87%)	0.2058
		T	0.2275	CT	69 (38.76%)	
				TT	6 (3.37%)	
	rs333^a^	WT	0.9944	WT/WT	177 (98.88%)	1.0000
		Δ32	0.0056	WT/Δ32	2 (1.12%)	
				Δ32/Δ32	0	

^a^ one sample failed to genotype.

^b^ two samples failed to genotype.

**Table 4:** Association of genotype with transmission.

Gene	SNP	Additive		Recessive		Dominant	
		Chi square	*P*	OR (95% CI)	*P*	OR (95% CI)	*P*
*CXCL12*	rs1801157	0.00	0.99	2.18 (0.3, 24.73)	0.43	0.9 (0.46, 1.76)	0.75
*CCR2*	rs1799864	1.02	0.31	>10 (1.3, >10)	0.01	1.07 (0.56, 2.02)	0.88
*CCR5*	rs2856758	1.88	0.17	NA		0 (0, 5.68)	0.50
	rs2734648	0.59	0.44	1.09 (0.51, 2.31)	0.86	0.57 (0.25, 1.26)	0.14
	rs1799987	0.34	0.56	1.31 (0.49, 3.6)	0.65	1.13 (0.58, 2.22)	0.75
	rs1799988	0.62	0.43	1.46 (0.55, 3.95)	0.50	1.16 (0.6, 2.29)	0.64
	rs41469351	NA		NA		NA	
	rs1800023	0.43	0.51	1.68 (0.75, 3.8)	0.19	0.9 (0.43, 1.91)	0.86
	rs1800024	1.68	0.20	>10 (1.3, >10)	0.01	1.18 (0.62, 2.23)	0.65
	rs333	1.9	0.17	NA		0 (0, 5.62)	0.5

**Table 5:** Association of haplotype with transmission.

Haplotype^a^	Name	Frequency	OR (95% CI)	*P*
hGAGGTCACI	HHA	0.08	1.49 (0.5, 2.36)	0.84
hGATGTCGCI	HHC	0.50	1.27 (0.8, 2.02)	0.30
hGAGACCACI	HHE	0.17	1.34 (0.58, 1.86)	0.89
hAAGACCATI	HHF*2	0.21	1.31 (0.41, 1.2)	0.20

^a^ The rs333 CCR5 Δ32 genotype was coded as I (insertion)/D (deletion).

### Clinical, Social and Genetic Factors Related to HIV Transmission

The two most significant factors affecting transmission, change in condom use and ART were independent when assessed in a multivariable model. The strength of the model increased when sex of the index partner was included as a random effect in a linear mixed model (Table 6), therefore sex of index partner was used as a random effect for modeling the genetic terms when assessed in multivariable models including these factors. Education was independent of treatment as a predictive factor, but was not independent of condom use. rs1799864 and rs1800024 both retained significance when tested with change in condom use or ART, however it should be noted that few patients in this study were untreated (Table 1), and no untreated patients were homozygous for the minor allele at rs1799864 and rs1800024. When condom use, ART, and rs1799864 or rs1800024 are all included in a single model, then only condom use retained significance.

**Table 6:** Analyses of Clinical, Social and Genetic Factors Related to Transmission.

		OR (95% CI)	*P*
	Antiretroviral therapy^a^	0.81 (0.63, 1.05)	0.1122
	Change in condom use after index diagnosed^b^	1.14 (1.03, 1.28)	0.0154
**Stratified by gender**	Antiretroviral therapy^a^	0.81 (0.67, 0.99)	0.0453
	Change in condom use after index diagnosed^b^	1.14 (1.08, 1.21)	<.0001
	Education level (index)	0.87 (0.79, 0.95)	0.0032
	Antiretroviral therapy^a^	0.81 (0.67, 0.98)	0.0318
	Education level (index)	0.92 (0.82, 1.02)	0.1064
	Change in condom use after index diagnosed^b^	1.14 (1.07, 1.21)	<.0001
	Change in condom use after index diagnosed^b^	1.16 (1.09, 1.23)	<.0001
	rs1800024 (recessive model)^c^	1.91 (1.03, 3.55)	0.0421
	Change in condom use after index diagnosed^b^	1.15 (1.09, 1.22)	<.0001
	rs1799864 (recessive model)^d^	1.9 (1.02, 3.55)	0.0464
	Antiretroviral therapy^a^	0.8 (0.66, 0.97)	0.023
	rs1800024 (recessive model) ^c^	1.64 (1.1, 2.45)	0.0155
	Antiretroviral therapy	0.8 (0.66, 0.97)	0.0229
	rs1799864 (recessive model) ^d^	1.64 (1.1, 2.45)	0.0155
	Antiretroviral therapy^a^	0.83 (0.68, 1.01)	0.0692
	Change in condom use after index diagnosed^b^	1.15 (1.09, 1.22)	<.0001
	rs1800024 (recessive model) ^c^	1.85 (1, 3.41)	0.0525
	Antiretroviral therapy^a^	0.82 (0.68, 1)	0.0566
	Change in condom use after index diagnosed^b^	1.15 (1.08, 1.21)	<.0001
	rs1799864 (recessive model) ^d^	1.83 (0.99, 3.4)	0.0579

^a^ Non-treated vs treated.

^b^ Change in condom use was measured as ‘frequency of use after diagnosis—‘frequency of use before diagnosis, with use coded as: 1 = every sexual encounter, 2 = frequent use (the majority of sexual encounters), 3 = infrequent use (minority of sexual encounters), and 4 = never used.

^c^ TT vs non-TT.

^d^ AA vs non-AA.

## Discussion

In this study we found that several social and genetic factors can influence transmission rate of HIV between serodiscordant couples. As part of a large cohort of former plasma donors infected with HIV in Henan province, a subset of cohabitating partners was selected for analysis of transmission risk factors. Education levels, condom use, anti-retroviral treatment of the infected partner, and frequency of sexual activity all affected transmission. Change in condom use and ART were of the greatest significance and retained independence when assessed together. This is similar to other findings which have seen that condom use [20,21] and frequency of sexual activity are strongly associated with risk of transmission [20]. It has been reported that ART for HIV-1 infected individuals decreased transmission risk and the WHO recommends ART for index partners within serodiscordant couples to prevent HIV transmission [22,23]. Additionally, a recent national observational cohort study showed that ART for index partners in serodiscordant couples reduced HIV transmission in China [24]. However, this study found that ART was associated with increased risk of transmission. This difference is likely due to several reasons of this study not being a controlled trial for the efficacy of ART in reducing transmission; patients selected for treatment in this cohort exhibited relatively high viral loads, disease progression at clinical stages 3/4, or CD4 T cell count <200/mm^3^, all of which can increase the likelihood of transmission. A previous study has shown that higher viral load increased the risk of heterosexual transmission of HIV [25]. In addition, more clinical details of treatment time, ART regimens and compliance of index partners should be taken into consideration in the study of ART and HIV transmission.

Divergent results for the relationship of education to HIV transmission have been found in previous studies. A positive relationship between education level and HIV rates has been observed [26,27], some studies found no significant association [28,29] and other studies suggest that a significant inverse relationship can also exist [30,31]. In this study we found a significant negative association between transmission and education. This predictor was not independent of condom use, concomitantly, education and change in condom use after diagnosis had a strong positive relationship (*P* = 0.0041) in a linear regression model. This suggests that a primary mechanism through which education allows lower transmission in this cohort is through awareness that condom use decreases likelihood of infection.

No association was seen between viral load and transmission, though other studies have reported significant associations [25,32,33]. However, it has been seen in one study that transmission did not occur when viral copy numbers were below 1500 copies per ml [25], and in this study only 48 participants had viral loads greater than 1500. It is likely that the study presented here was underpowered to identify an association between viral load and transmission.

Several *CCR5* mutations, one *CCR2* and one *CXCL12* mutation were assessed for their association with transmission of HIV. The *CCR2* mutation rs1799864 and the *CCR5* variant rs1800024 showed a significant association with transmission in this study and retained significance in the presence of condom use and treatment. The *CCR2* variant is a conservative mutation (V to I) occurring within the first transmembrane domain of the receptor, while the *CCR5* variant is a C to T mutation in an intron. The two alleles have been reported to be in strong linkage disequilibrium, as was also seen in this study [34]. These two variants (represented by the HHF*2 haplotype) have been seen to associate with lower viral loads in an African population but did not have a significant association with transmission [3], and a significant association with decreased transmission was observed in a Columbian cohort [35]. However, a study in a Chinese population found a significant association with increased transmission for the *CCR5* rs1800024 variant and a non-significant but similarly oriented association for the *CCR2* allele [36]. It is very likely that the genetic background in different ethnic groups is a strong modifier on the association of these variants with risk of transmission, and should be studied in greater depth.

This study relies partly on surveys of the participants, and thus a limitation is the accuracy of responses. This can be especially acute when social stigmas are attached to the questions. For this study, though the partners were questioned independently, there was a high degree of correspondence between responses. For all queries on sexual activity and condom use before and after infection there was an average correlation between index and exposed partners of 0.87. However for the question on condom use before infection, the correlation between partners was only 0.78, with the exposed partner claiming a higher use of condom use than the infected partner (Table 2), possibly due to a desire to not admit to risky behavior. However, the change in condom use of the index partner was a stronger factor in predicting transmission than that as reported by the exposed partner, so the index partner’s response was used for multivariable analyses.

Another limitation of this study was the sample size, in particular among untreated cases, due in part to free screening and treatment plans provided by the provincial government, coupled with the rarity of some the studied alleles. Increased confidence in these results will entail gathering survey and genotype data from a larger population.

Two mutations in *CCR2* and *CCR5*, rs1799864 and rs1800024, were found to predict HIV transmission. These two variants were tightly linked, with a correlation of the genotypes of 0.91. As these variants were independent of treatment and condom use, either variant may serve to help stratify individuals by risk for HIV transmission. Risk stratification can be useful in monitoring programs, such as that employed in Henan province, to efficiently allocate limited resources to those most at need and to reduce rates of false positives in screening programs. Further studies are intended follow-up these results.

## Supporting Information

S1 Fig *CCR2* and *CXCL12* mutations. The variant sites assessed in this study are shown mapped to their genomic positions. (TIF)

S1 Table Primers design for genotyping. (DOC)
