# Supplementary figures and images for: Analysis of Social and Genetic Factors Influencing Heterosexual Transmission of HIV within Serodiscordant Couples in the Henan Cohort
Source: PLoS One. 2015 Jun 11;10(6):e0129979. doi: 10.1371/journal.pone.0129979 (PMC4465854; doi:10.1371/journal.pone.0129979)

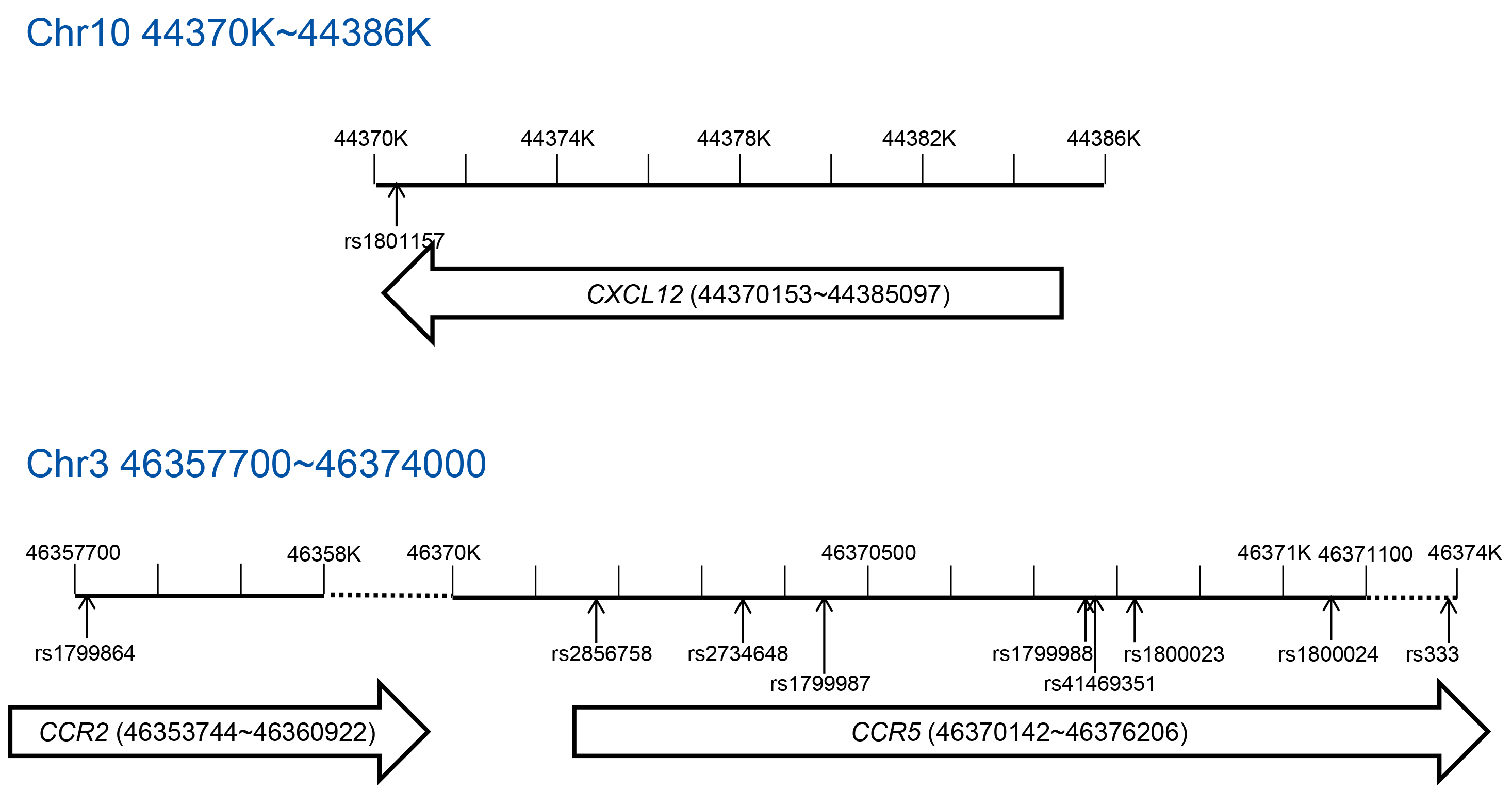

Supplement: S1 Fig — (TIF) [file pone.0129979.s001.tif]
