# Supplementary material for: Analysis of Social and Genetic Factors Influencing Heterosexual Transmission of HIV within Serodiscordant Couples in the Henan Cohort
Source: PLoS One. 2015 Jun 11;10(6):e0129979. doi: 10.1371/journal.pone.0129979 (PMC4465854; doi:10.1371/journal.pone.0129979)
Supplement: S1 Table — (DOC) [file pone.0129979.s002.doc]

**S1 Table. Primers design for genotyping.**

| **SNP** | **Primer^a^** | **Primer sequence** |
| --- | --- | --- |
| rs1801157 | F | TGGAGAGCCACCAAGAGGGA |
|  | R | TGAGCAGAACGTGGAGGATGT |
| rs1799864 | F | TACCAACGAGAGCGGTGAAGAAG |
|  | R | CATTGCATTCCCAAAGACCCACTC |
| rs2856758/rs2734648/ rs1799987 | F | CAGATGTCACCAACCACCAAGAG |
|  | R | GCTCATCCCACTACACAGAATCTG |
| rs1799988/rs41469351/ rs1800023/ rs1800024 | F | GACTAGATGAATGTAAATGTTCTTCTAGC |
|  | R | CGTCTGAAACTCATTCCAAACTGT |
| rs333 | F | CTGTCGTCCATGCTGTGTTTG |
|  | R | CTGGAAGGTGTTCAGGAGAAGG |

^a^F: Forward Primer; R: Reverse primer.
